# Metagenomic Insights into the Microbial Composition and Functional Potential of Cocoa (*Theobroma cacao *L.) During Fermentation and Drying in Colombia

**DOI:** 10.1007/s00248-026-02704-7

**Published:** 2026-03-10

**Authors:** Daniel López-Puentes, Zaida Zarely Ojeda-Pérez, Diana Marcela Arias-Moreno

**Affiliations:** 1 Grupo de Investigación (BIOPLASMA), Facultad de Ciencias Básicas. Escuela de Ciencias Biológicas, Universidad Pedagógica y Tecnológica de Colombia - UPTC, Tunja, Boyacá, Colombia; 2 Grupo de Investigación de Ecuaciones Diferenciales, Modelación y Simulación (GEDMyS), Facultad de Ciencias Básicas, Escuela de Matemáticas y Estadística, Universidad Pedagógica y Tecnológica de Colombia - UPTC, Tunja, Boyacá, Colombia; 3 Grupo de Investigación en Biotecnología y Producción Agrícola (GIBPA), Facultad de Ingeniería Agronómica, Departamento de Producción y Sanidad Vegetal, Universidad del Tolima, Ibagué, Colombia

**Keywords:** Metagenomics, Cocoa, Fermentation, Drying, Composition, Pathway

## Abstract

Shotgun metagenomics is an approach increasingly applied to investigate microbial succession and functional potential in complex fermented food systems, including cocoa bean fermentation. In this study, we used Illumina-based shotgun metagenomic sequencing to characterize microbial community dynamics and metabolic potential across two post-harvest cocoa processing routes (R1 and R2) in Boyacá, Colombia, encompassing both fermentation and drying stages. Cocoa beans were sampled at defined time points during fermentation and subsequent natural drying, and non-host metagenomic reads were subjected to taxonomic classification and functional annotation to assess fungi, bacteria, and viruses. A clear multi-ecological succession was observed throughout post-harvest processing. Fungal communities shifted from a yeast-dominated profile, mainly *Saccharomyces* and *Pichia* during fermentation, to the emergence of the filamentous fungus *Aspergillus* during drying. Bacterial populations transitioned from diverse *Enterobacteriaceae* in early fermentation to a near-complete dominance of *Acetobacter*, which persisted throughout the drying phase. Viral communities also displayed structured successional patterns, with *Lambdavirus* and *Punavirus* prevalent in early fermentation, followed by *Spbetavirus*, *Lafunavirus*, and *Pemunavirus* during later stages and drying. Functional analyses revealed high metabolic potential for carbohydrate, energy, and amino acid metabolism during early fermentation, followed by a marked reduction in later stages, indicating a metabolic slowdown. Core metabolic functions were retained during drying at substantially lower activity levels. This integrated metagenomic analysis links microbial structure to functional potential and provides a scientific basis for optimizing starter cultures and post-harvest processing strategies to enhance cocoa quality and safety.

## Introduction

Metagenomics, particularly shotgun sequencing, has proven to be a powerful tool for investigating microbial communities in fermented foods. This approach facilitates the comprehensive characterization of bacterial populations, their metabolic potential, and genetic features throughout fermentation processes [1–3]. Research has highlighted the dominance of lactic acid bacteria (LAB) genera such as *Leuconostoc*, *Lactiplantibacillus*, and *Weissella* in various fermented foods [1, 4, 5]. Additionally, metagenomics has enabled the identification of probiotics, pathogenic bacteria, and antimicrobial resistance genes in traditional fermented foods [4]. This technology has provided valuable insights into key metabolic pathways involved in fermentation, including those related to carbohydrate metabolism [4]. With advancements in sequencing technologies making them increasingly accessible and accurate, metagenomics is being more widely applied to fermented food studies, opening new possibilities for food waste management and the development of enhanced traits in fermented products [6].

On the other hand, cocoa bean fermentation is a complex microbial process crucial for chocolate production. It involves a succession of yeasts (Y), lactic acid bacteria (LAB), and acetic acid bacteria (AAB) [7, 8]. The fermentation typically begins with yeasts and LABs consuming sugars, followed by AAB oxidizing ethanol and lactic acid [7]. This process produces key metabolites like ethanol, lactic acid, and acetic acid, which contribute to flavor development [9]. Fermentation and drying are critical processes in cocoa production, as they significantly influence its sensory and chemical properties. Studies conducted in Colombia have demonstrated that these processes affect the levels of polyphenols, anthocyanins, and sugars in cocoa beans [10, 11]. An optimal fermentation duration of approximately six days, followed by drying at 70 °C, has been shown to produce desirable volatile compounds while minimizing the development of off-flavors [12]. Additionally, the genetic composition of cocoa cultivars plays a crucial role in determining the fermentation dynamics and final product quality [11]. Recent research in Colombia has identified significant variations in polyphenol content, flavonoid concentrations, and antioxidant activity across different fermentation sites [13]. These findings underscore the necessity of meticulously managing fermentation and drying processes to achieve high-quality cocoa, particularly given Colombia’s potential in the premium cocoa market.

Moreover, metagenomic analyses of cocoa bean fermentation have revealed diverse microbial communities dominated by (Y), (LAB), and (AAB). Key genera and families include *Saccharomyces* (Y), *Lactobacillaceae* (LAB), and *Acetobacter* (AAB), which contribute to the fermentation process through carbohydrate metabolism and the production of organic acids [14]. These microorganisms play essential roles in the development of flavor precursors and aroma compounds critical for cocoa quality [15]. Some studies identified a core set of dominant microorganisms shared among different farms, suggesting the potential for standardized fermentation processes [16]. Functional metagenomic analyses have further elucidated the metabolic capabilities of bacterial communities, including stress responses in acetic acid bacteria (AAB) [17]. This knowledge can inform the development of targeted starter cultures and the optimization of fermentation processes to enhance cocoa quality [14, 17]. Notably, some studies have combined metagenomic and metatranscriptomic approaches to comprehensively characterize microbial diversity and metabolic activity during cocoa fermentation, these studies have revealed previously overlooked microbial species and metabolic functions that could be leveraged to design tailored starter cultures [14, 18].

The research demonstrates two key metabolic phases during spontaneous fermentation: an exothermic phase with high metabolic activity and an isothermic phase with reduced activity [19]. A core microbiome of nine dominant microorganisms was identified across different farms, including key species like *Limosilactobacillus fermentum* and *Saccharomyces cerevisiae* [16]. Metabolomic analyses identified 25 differential metabolites during post-harvest processing, with significant variations in peptides, sugars, amino acids, and phenolic compounds [20].

Recent research has advanced understanding of cocoa fermentation through metabolomics and omics approaches, revealing complex biochemical processes that influence chocolate quality. Metabolomics enables detection of key metabolites responsible for taste, aroma, and quality formation during spontaneous fermentation, allowing characterization of different fermentation stages and optimization of processing time [21]. Comprehensive omics studies demonstrate clear metabolite differences based on geographical origin, cocoa type, and processing stage, while identifying recurrent microbial species including *Candida*, *Pichia*, *Lactobacillus*, *Acetobacter*, and *Bacillus* [22]. Next-generation sequencing technologies have revealed 99 previously unreported microbial genera and species in cocoa fermentation, with species composition varying significantly between cocoa-producing regions including Colombia [23]. Additionally, fermentation contributes to bioactive peptide formation through protein hydrolysis, potentially offering health benefits beyond traditional polyphenol compounds [24].

Some studies examine genetic diversity and quality characteristics of cocoa across different regions, with limited specific focus on Boyacá, Colombia. Colombian cocoa demonstrates significant genetic diversity, with collections showing high expected heterozygosity (HE = 0.314) and classification into distinct genetic groups including Criollo, Amelonado, and Upper Amazon Forastero types [25, 26]. Regional variations in cocoa quality are evident, as demonstrated in southern Colombia where specific clones like ICS-95 and TSH-565 exhibited superior sensory attributes including floral, sweet, and fresh fruit notes [27]. Genetic classification systems identify materials as Modern Criollo, Forasteros, and Trinitarios based on molecular markers and quality profiles [26]. While these studies establish frameworks for distinguishing cocoa varieties through genetic, sensory, and regional approaches, none specifically address Boyacá’s cocoa metagenomics characteristics (including viral components), suggesting a research gap for this particular Colombian region.

By investigating microbial diversity and functional capabilities during this critical stage of cocoa production, these studies elucidate the structure, functional annotation, and metabolic pathways of the cocoa microbiome. They provide valuable insights into fermentation processes that contribute to the formation of desirable flavor and aroma compounds [28, 29]. Furthermore, metagenomic analyses have been employed to explore microbial interactions and biochemical processes, aiming to optimize fermentation conditions and mitigate undesirable flavor outcomes in fine-flavor cocoa [16]. High-resolution functional metagenomic analysis of a single spontaneous cocoa bean fermentation sample has facilitated a deeper understanding of the metabolic capabilities and roles of the major microbial communities within this ecosystem [17]. Notably, this investigation represents one of the first metagenomic studies of cocoa bean fermentation in this region, providing a more comprehensive view of microbial diversity (fungi, bacteria and virus) compared to previous methodologies. It also highlights the limitations of both culture-dependent and culture-independent techniques in fully capturing the spectrum of microbial members and associated microorganisms. This study aims to explore the microbial community composition, structure and identify some functional potential of two cocoa fermentations and drying routes used in Colombia-Boyacá. While previous studies have characterized the cocoa microbiome, few have conducted shotgun metagenomic analyses to describe the complete microbial succession (fungi, bacteria, and viruses) across both fermentation and drying stages simultaneously, particularly in Colombian production systems with distinct environmental and operational conditions.

Specifically, we aimed to:

(i) Describe the microbial composition between fermentation routes with different drying and fermentation practices;
(ii) Identify functional pathways potentially related to cocoa fermentation and drying during post-harvest processing.

## Methodology

### a. Fermentation and Drying

**Fig. 1:**
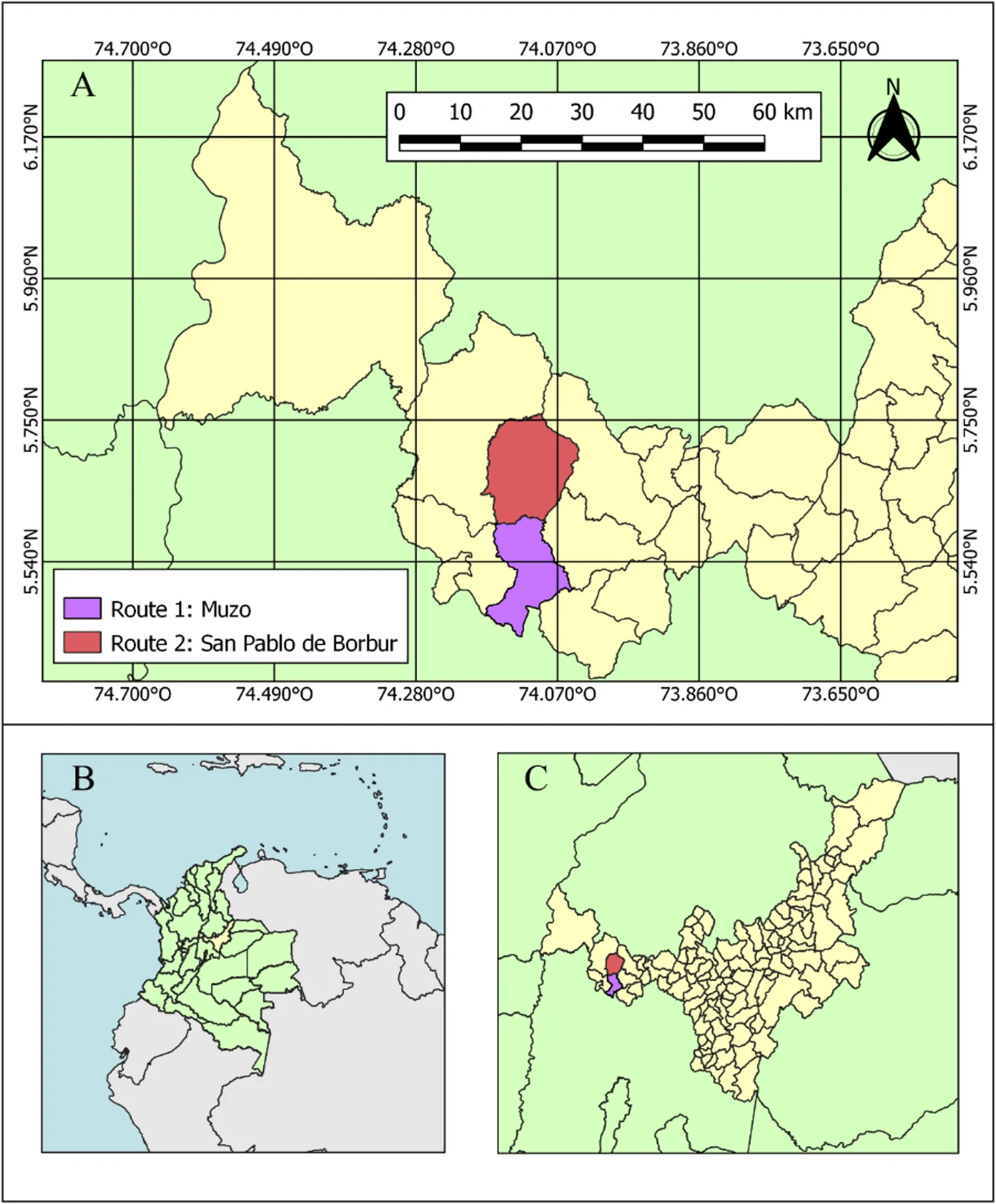
Geographical representation of sampling location routes (Route 1: R1 and Route 2: R2) for the fermentation and drying process of Colombian cocoa, Boyacá - department

The initial plant material consisted of mixtures of mature cocoa beans of *Criollo* and *Trinitario* TCS (Trinitario Colombia Selection, TCS-01 to TCS-19) origin sourced from various cocoa crops in the municipality of Muzo (Route 1) and San Pablo de Borbur (Route 2), Boyacá department, Colombia (Fig. 1). The ripe cocoa pods from these different crops’-origins were harvested during the primary harvest season in January 2022. The harvested cocoa underwent a 14-hour draining phase before the fermentation process began. A total of 800 kg of cocoa was evenly distributed across six wooden boxes, each with a capacity of 350 kg. Fermentation was conducted over 168 h, with daily turning to promote aeration and ensure uniform fermentation. Afterward, the cocoa beans were transferred to a greenhouse and spread on drying racks to initiate the drying process. Beans from each box were divided among four drying racks, which were randomly arranged to maintain sampling independence. This natural drying process lasted approximately seven days, during which the beans were periodically moved to reduce moisture content and minimize the growth of external fungi on the racks. The end of fermentation was determined using a cut test following standard cocoa post-harvest evaluation practices. Briefly, a random subsample was longitudinally cut and visually assessed. Fermentation was considered complete when the majority of beans exhibited a brown to dark-brown cotyledon color, indicating adequate internal fermentation, corresponding to at least ~ 70% well-fermented beans. Drying was conducted under natural greenhouse conditions following local post-harvest practices. The end of the drying stage was defined using a combination of operational and instrumental criteria. Operationally, beans were considered dry when no surface moisture was observed, indicating suitability for storage and transport. Instrumentally, drying was considered complete when bean moisture content reached values below 7.5%. Physicochemical data were not included.

In this study, an additional early fermentation sample (R1_Fer2) was intentionally included only for Route 1 to provide a more detailed characterization of the initial stages of microbial succession in at least one processing route. This sampling strategy was adopted to capture earlier ecological transitions during fermentation and to offer a more comprehensive temporal resolution for Route 1. A corresponding second early time point was not collected for Route 2, as the experimental design prioritized balanced sampling at later fermentation stages common to both routes. Additionally, the end of the drying stage differed between routes, with drying completion defined operationally and by instrumental moisture measurements at day 14 for Route 1 and day 16 for Route 2, reflecting differences in local drying conditions and practices.

### b. Field Experimental Design

The experimental design consisted of two blocks: one for Route 1 and one for Route 2. Each block contained different fermentation boxes, sampled at distinct time points: Samples for metagenomics analysis were collected from each route at specific time points. During fermentation, samples were taken only at 48, 72 h and 168 h (Days 2, 3 and 7). During the drying process, samples were similarly collected at specific time points: 24 h and 336 h (Days 1 and 14) for Route 1, and 24 h and 384 h (Days 1 and 16) for Route 2. All samples were taken approximately 20 cm below the surface at the center of the fermenting and drying cocoa mass. Samples were collected within the fermentation and drying mass of each wooden box. From this, samples were stored at −20 °C in sterilized plastic bags for shotgun metagenomics.

### c. Genomic DNA Extraction and Illumina Sequencing

For DNA extraction, the DNeasy^®^ Plant Mini Kit (QIAGEN^®^) was used following the manufacturer’s instructions. The quality and quantity of the extracted DNA were quantified using the Qubit™ fluorometer (Thermo Fisher Scientific Inc.) and the NanoDrop™ spectrophotometer (Thermo Fisher Scientific Inc.). Subsequently, the extracted DNA was stored at − 20 °C for further analysis. After confirming DNA integrity, sequencing libraries were constructed using the TruSeq^®^ Nano DNA Library Prep Kit (Illumina^®^). Library preparation was performed following Illumina^®^’s protocol for DNA samples, TruSeq^®^ Nano DNA Sample Preparation Guide (Part # 15041110 Rev. D). The prepared libraries were sequenced using the Illumina^®^ sequencing platform (Metagenome Shotgun Sequencing), specifically designed for paired-end sequencing, with a read length of 151 base pairs, Illumina^®^ System. Accession: PRJNA1264670 ID: 1,264,670. https://www.ncbi.nlm.nih.gov/bioproject/1264670.

### d. Bioinformatics Analysis

The bioinformatic methodology was based on a reproducible Bash pipeline executed under Linux using Python v3.13.5, with 20 threads, 64 GB of RAM, and an Intel Core Ultra 7 (256 K) processor. Initially, a quality control of raw paired-end reads was performed using FastQC v0.11.9 [30] and summarized with MultiQC v1.31 [31], assessing parameters such as per-base quality, GC content, and adapter contamination. Reads were then trimmed and filtered with Trimmomatic v0.36 [32], removing low-quality regions and Illumina adapter sequences. Clean reads were mapped against the (*Theobroma cacao* L.) reference genome using Bowtie2 v2.4.4 [33], and unmapped (non-host) reads were extracted with Samtools v1.13 [34] to eliminate host-derived contamination. A second quality assessment was conducted on these non-host reads with FastQC v0.11.9 [30] and MultiQC v1.31 [31]. Taxonomic classification was performed using Kraken2 [35] and Bracken [36] with specific databases for fungi, bacteria, and viruses. Subsequently, non-host reads were assembled into contigs using metaSPAdes v4.2.0 [37] with *k-mer* sizes of 55, 75, and 95, retaining contigs ≥ 500 bp. Assembly quality was evaluated using metaQUAST v5.0.2 [38], employing NCBI 16 S rRNA sequences as reference, and additionally by remapping non-host reads to their respective assemblies with Bowtie2 [33] to determine alignment efficiency. Finally, gene prediction and functional annotation were conducted with Prokka [39] in *--metagenome* mode, generating annotated files for each assembled sample. Functional categories were reconstructed using the KEGG database [40] to identify potential metabolic pathways and biological processes represented within the metagenomes. All graphical visualizations were generated in RStudio (v 2025.09.1–401) using the ggplot2 and RColorBrewer packages for data plotting and color palette management.

## Results

**Fig. 2:**
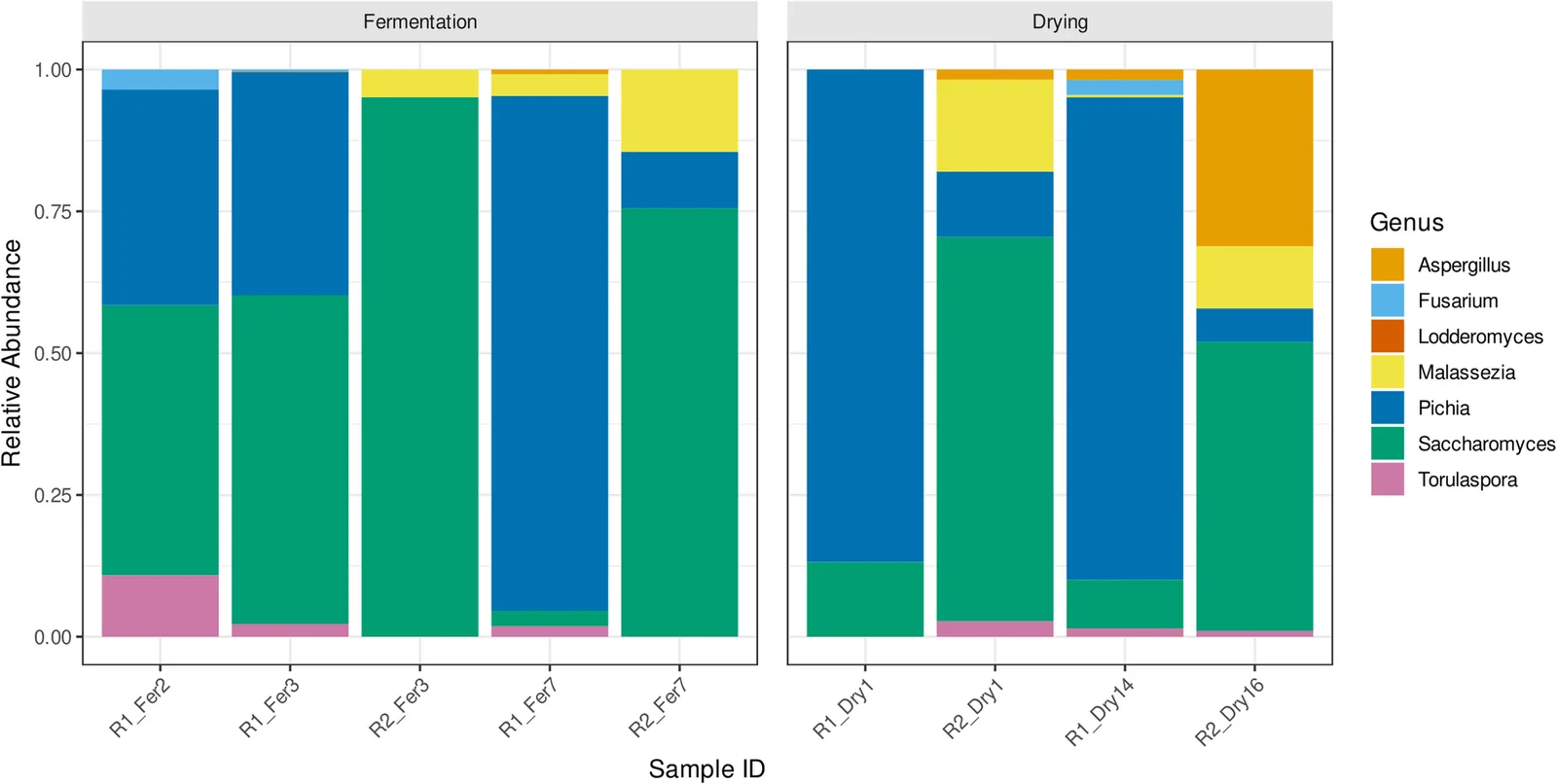
Bar plot showing the relative abundance (%) of the predominant fungal genera across two post-harvest processing routes (R1 and R2) at different time points during fermentation (Fer) and drying (Dry), based on shotgun metagenomic sequencing data. An additional early fermentation sample (R1_Fer2) was included exclusively for Route 1 to provide higher temporal resolution of initial fungal succession; a corresponding second early fermentation time point was not collected for Route 2. Therefore, direct route-to-route comparisons are based on shared sampling points. Fungal community structure shifted markedly between fermentation and drying phases. Early and mid-fermentation stages were dominated by the yeasts *Saccharomyces* and *Pichia*, with route-dependent relative abundances. A pronounced ecological shift occurred during drying, characterized by a decline of these yeasts and the emergence of the filamentous fungus *Aspergillus*, particularly in the later stages of Route 2. Drying was considered complete at day 14 for Route 1 and day 16 for Route 2, reflecting route-specific processing conditions. Sample names indicate route (R1 or R2), processing stage, and day of collection

Initially, a total of nine cocoa samples were analyzed across two post-harvest processing routes (R1 and R2), covering fermentation and drying stages. The relative abundance profiles revealed clear dominance patterns among yeast genera, and the abundance of fungal genera varied significantly across stages and routes (Fig. 2). During fermentation, *Saccharomyces* and *Pichia* were the dominant genera in Route 1, ranging from 47.6 to 57.9% and 38.0–39.3%, respectively, while *Torulaspora* and *Fusarium* appeared in minor proportions (< 10.9%). In contrast, Route 2 fermentation was characterized by a strong predominance of *Saccharomyces* (95.1% in R2_Fer3), with a smaller contribution from *Malassezia* (4.9%). At later fermentation stages (R1_Fer7 and R2_Fer7), *Pichia* reached its highest abundance in Route 1 (90.7%), while in Route 2 it decreased to 9.9%, a stage where *Saccharomyces* was dominant at 75.6%. During the drying process, *Pichia* remained the predominant genus in Route 1 (ranging from 86.8% to 85.0% in R1_Dry1 and R1_Dry14, respectively) and was moderately represented in Route 2 (11.5% and 5.9%). Conversely, *Saccharomyces* exhibited variable abundances in Route 2 drying samples (67.8% in R2_Dry1 and 51.0% in R2_Dry16). *Aspergillus* was detected almost exclusively during the drying phase, peaking at 31.2% in R2_Dry16, while *Malassezia* also persisted at low to moderate levels (≤ 16.2%). Other genera such as *Torulaspora*, *Fusarium*, and *Lodderomyces* appeared sporadically with negligible relative abundances (< 3.6%).

**Fig. 3:**
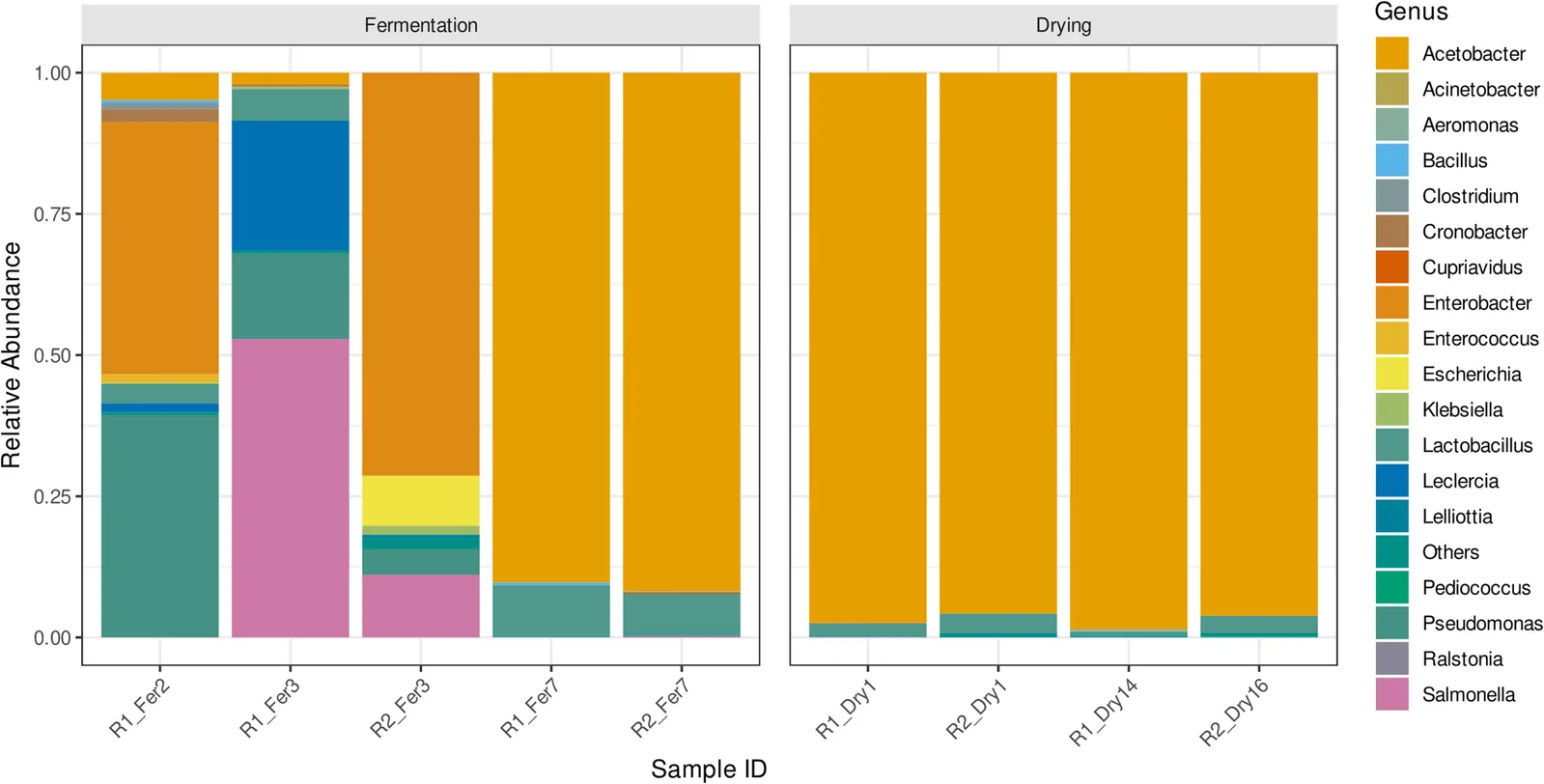
Bar plot depicting the relative abundance of bacterial genera across two processing routes (R1 and R2) at different fermentation (Fer) and drying (Dry) time points, as determined by shotgun metagenomic sequencing. An early fermentation sample at day 2 (R1_Fer2) was included only for Route 1 to better characterize early microbial succession, whereas Route 2 was sampled starting at day 3 of fermentation. Accordingly, comparative analyses between routes focus on shared time points. Early fermentation was characterized by a diverse bacterial assemblage dominated by *Enterobacteriaceae* (e.g., *Enterobacter*, *Salmonella*) and *Pseudomonas*. A clear successional transition occurred during later fermentation, resulting in near-complete dominance of *Acetobacter*, which persisted throughout drying. *Lactobacillus* remained a consistent secondary inhabitant. Drying endpoints differed between routes (day 14 for R1 and day 16 for R2) and are indicated accordingly

Subsequently, the bacterial succession during fermentation and drying followed a distinct trajectory, characterized by an initial dominance of *Enterobacteriaceae*, which was subsequently replaced by a community overwhelmingly dominated by acetic acid bacteria AAB (Fig. 3). In the early stages of fermentation (R1_Fer2, R2_Fer3), the bacterial community was diverse and dominated by genera from the *Enterobacteriaceae* family, including *Enterobacter* (44.7% in R1_Fer2; 71.4% in R2_Fer3) and *Salmonella* (52.9% in R1_Fer3; 11.1% in R2_Fer3). *Pseudomonas* (39.4% in R1_Fer2). *Lactobacillus* was also present from the onset in Route 1 (3.5–5.6%). A decisive microbial shift occurred by the late fermentation stage (R1_Fer7, R2_Fer7), where *Acetobacter* emerged as the overwhelmingly dominant genus, constituting 90.3% and 91.9% of the community, respectively. This dominance of *Acetobacter* intensified and persisted throughout the entire drying phase in both routes, consistently accounting for > 95% of the relative abundance in all drying samples (ranging from 95.8% to 98.6%). *Lactobacillus* was the only other genus that persisted throughout all stages, though at much lower relative abundances (< 9%). All previously abundant *Enterobacteriaceae* genera became undetectable following the fermentation phase.

**Fig. 4:**
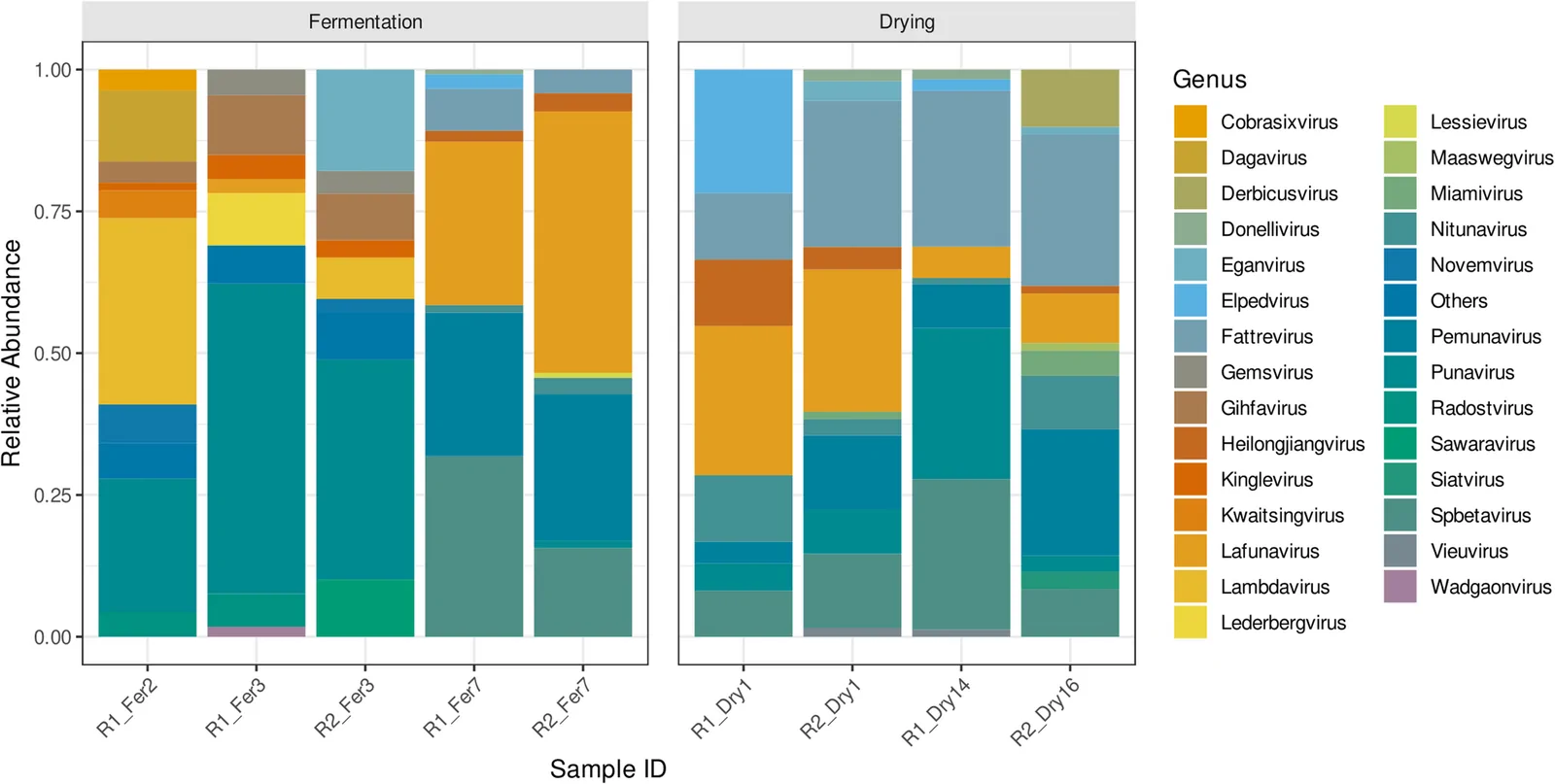
Bar plot illustrating the relative abundance (%) of viral genera identified through shotgun metagenomic sequencing across two processing routes (R1 and R2) during fermentation (Fer) and drying (Dry). The inclusion of an early fermentation sample (R1_Fer2) exclusively for Route 1 allowed enhanced resolution of early virome dynamics, while Route 2 sampling began at day 3 of fermentation. Early fermentation stages were characterized by diverse bacteriophage communities, including *Lambdavirus* and *Punavirus*. A distinct ecological transition occurred in later fermentation, with dominance shifting toward *Spbetavirus*, *Lafunavirus*, and *Pemunavirus*. This viral consortium persisted into the drying phase, showing dynamic changes such as the increase of *Fattrevirus* and the appearance of *Derbicusvirus* in late drying samples. Drying completion differed between routes (day 14 for R1; day 16 for R2), reflecting operational differences and is indicated in the sample labels

Then, the viral composition underwent a clear and quantitative successional pattern, shifting from a diverse community in early fermentation to a distinct, specialized consortium in later stages. Early fermentation was characterized by a high diversity of bacteriophages (Fig. 4). In R1_Fer2, *Lambdavirus* (32.9%) and *Punavirus* (23.5%) were dominant, while *Punavirus* also prevailed in R1_Fer3 (54.7%) and R2_Fer3 (38.8%). A definitive community shift occurred by late fermentation (R1_Fer7, R2_Fer7), where these early-stage viruses were largely displaced. A new viral ensemble emerged, dominated in R1_Fer7 by *Spbetavirus* (31.9%) and *Lafunavirus* (28.8%), and in R2_Fer7 by *Lafunavirus* (46.0%) and *Pemunavirus* (25.7%). This late-stage consortium persisted into the drying phase, though with dynamic fluctuations; for instance, *Fattrevirus* increased to 27.4% in R1_Dry14 and 26.6% in R2_Dry16, while *Punavirus* remained detectable in R1 (26.6% in R1_Dry14) but not in R2. Route-specific variations were evident, with *Derbicusvirus* appearing solely in R2_Dry16 at 10.1%. The drying phase presented a more stable but complex viral community compared to the highly dynamic fermentation stages.

**Fig. 5:**
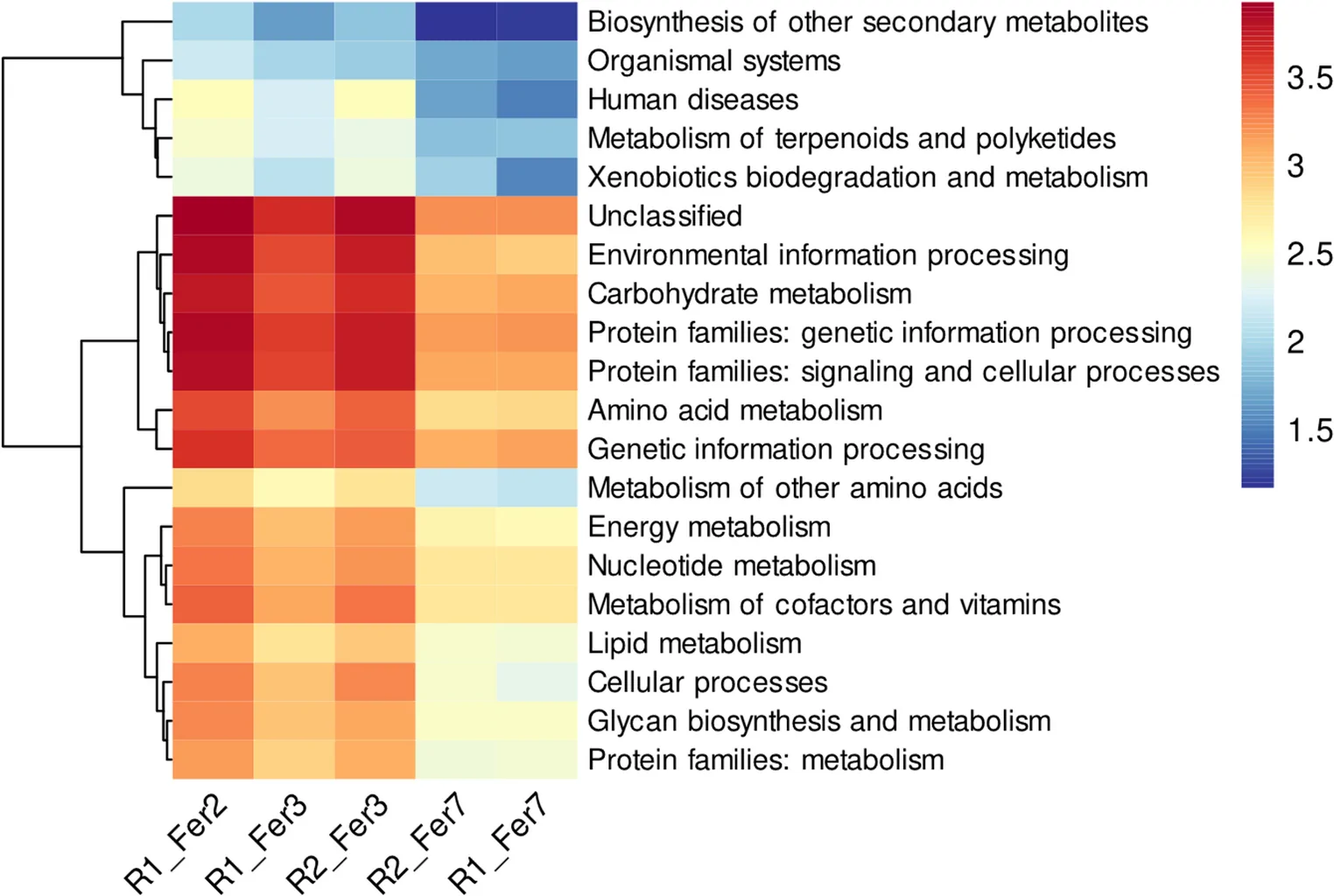
The heatmap displays the abundance of potential key metabolic pathways, based on KEGG annotation, across different fermentation time points (R1_Fer2, R1_Fer3, R2_Fer3, R1_Fer7, R2_Fer7). The inclusion of R1_Fer2 provides additional insight into early-stage metabolic activity for Route 1, while Route 2 fermentation profiles begin at day 3. Functional comparisons between routes are restricted to shared time points. The abundance values, represented as read counts, were transformed using $log_{10}(x+1)$ for visualization and are indicated by the color intensity scale. Each row represents a specific KEGG pathway category, and each column represents a fermentation sample. The gradient visually underscores the pronounced reduction in the abundance of major metabolic pathways—such as carbohydrate metabolism, environmental information processing, genetic information processing, signaling and cellular processes and others like amino acid metabolism—from the early to the late stages of fermentation, indicating a systemic decrease in overall microbial metabolic potential over time

Afterwards, the functional potential of the microbial communities, based on KEGG pathway annotation, revealed distinct metabolic dynamics across fermentation stages in both processing routes. The data, represented as $\:{log}_{10}(x+1)$ to visualize wide-ranging abundances, showed a significant reduction in metabolic activity as fermentation progressed (Fig. 5). In early fermentation samples, pathways related to Carbohydrate metabolism were highly abundant, with read counts of 6072 ($\:{log}_{10}$: 3.78) in R1_Fer2 and 4533 ($\:{log}_{10}$: 3.66) in R2_Fer3. Similarly, Environmental Information Processing [7042 reads ($\:{log}_{10}$: 3.85) in R1_Fer2] and Amino Acid Metabolism [3450 reads ($\:{log}_{10}$: 3.54) in R1_Fer2] were prominent, reflecting intense microbial activity for energy production and growth. A pronounced decline was observed in the later fermentation stages (R1_Fer7, R2_Fer7). Carbohydrate metabolism reads dropped to 1314 ($\:{log}_{10}$: 3.12) and 1164 ($\:{log}_{10}$: 3.07), respectively. This trend was consistent across most major categories; for instance, Energy Metabolism decreased from 1950 reads ($\:{log}_{10}$: 3.29) in R1_Fer2 to 399 reads ($\:{log}_{10}$: 2.60) in R1_Fer7. The log-transformed values, which compress the scale, clearly illustrate this widespread decrease in metabolic potential, suggesting a systemic metabolic slowdown in the fermentative microbial community, likely due to substrate depletion and the accumulation of inhibitory end-products. The analysis reveals a high functional potential in early fermentation stages, dominated by Carbohydrate Metabolism ($\:{log}_{10}$: 3.78), Environmental Information Processing ($\:{log}_{10}$: 3.85), Amino Acid Metabolism ($\:{log}_{10}$: 3.54), and Genetic Information Processing (log10: 3.65). Other highly represented categories included Metabolism of Cofactors and Vitamins ($\:{log}_{10}$: 3.41) and Nucleotide Metabolism ($\:{log}_{10}$: 3.33). A consistent and marked decrease in the abundance of virtually all these core metabolic and cellular processes is observed in later fermentation stages. This overall reduction highlights a systemic metabolic downshift in the microbial community as fermentation progresses.

**Fig. 6:**
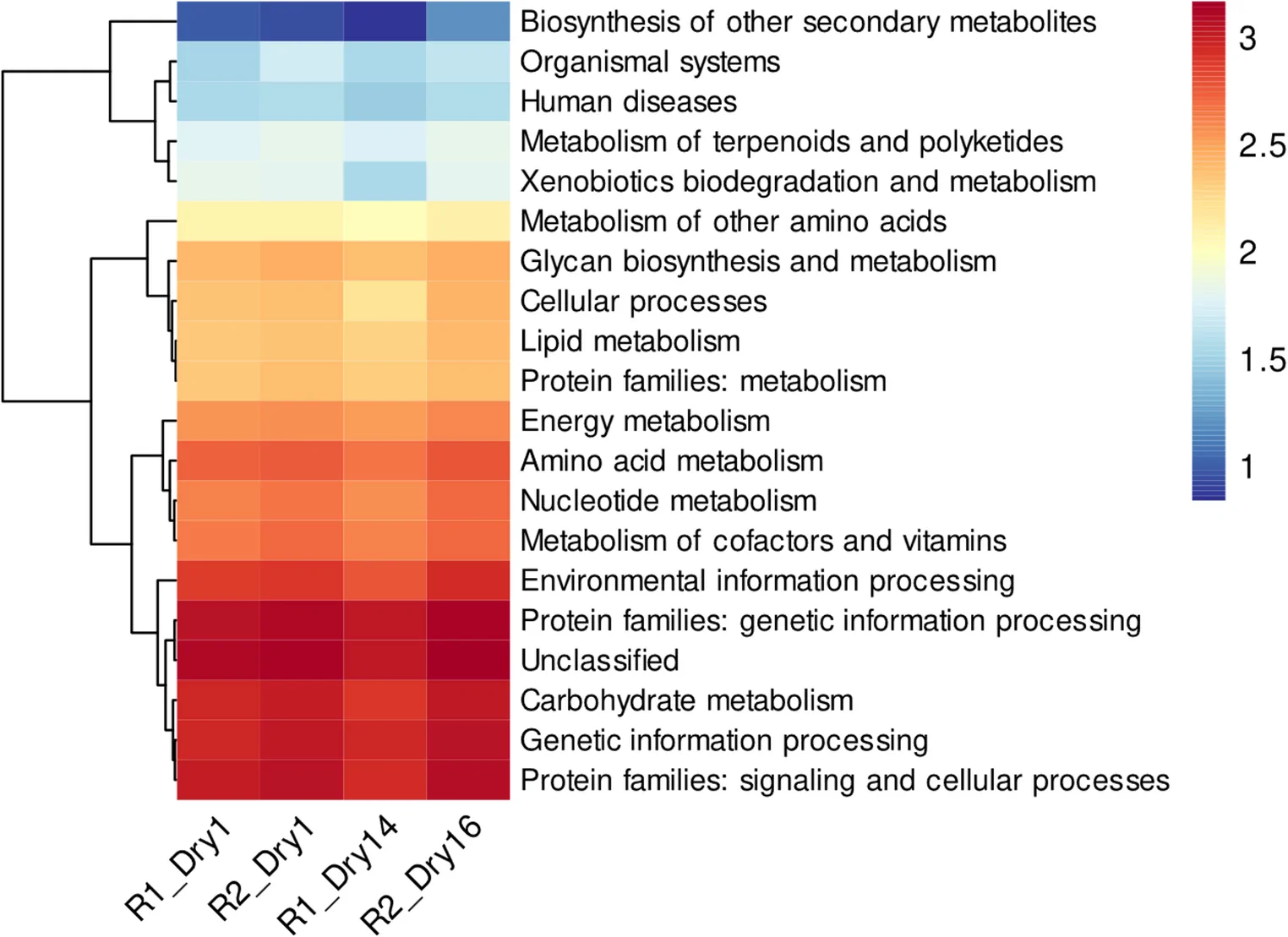
Heatmap visualization of KEGG pathway abundances across drying samples (R1_Dry1, R2_Dry1, R1_Dry14, R2_Dry16) shows the functional potential of microbial communities during this processing phase. Drying was considered complete at day 14 for Route 1 and day 16 for Route 2, based on route-specific post-harvest practices. Abundance values are represented as $log_{10}$ (read count + 1) and displayed on a color scale. The analysis reveals that Genetic Information Processing ($log_{10}$: 2.98–3.06) and Protein Families: Genetic Information Processing (log₁₀: 3.04–3.13) maintained the highest relative abundance throughout drying. Core metabolic pathways including carbohydrate metabolism ($log_{10}$: 2.91–3.04), amino acid metabolism ($log_{10}$: 2.66–2.80), and nucleotide metabolism ($log_{10}$: 2.68–2.73) showed consistent moderate abundance across all samples. The functional profiles remained remarkably stable between processing routes R1 and R2, with R2 samples generally exhibiting slightly higher abundance values

Ultimately, the functional annotation of microbial communities during the drying phase revealed a conserved metabolic profile characterized by sustained but reduced activity across core metabolic pathways compared to earlier fermentation stages (Fig. 6). The data, represented as $\:{log}_{10}(x+1)$ for standardized comparison, showed that Genetic Information Processing was the most abundant category across all drying samples [e.g., R2_Dry16: 1138 read ($\:{log}_{10}$: 3.06)], followed by Protein Families: Genetic Information Processing [R2_Dry16: 1349 read ($\:{log}_{10}$: 3.13)] and Carbohydrate Metabolism [R2_Dry16: 1094 read ($\:{log}_{10}$: 3.04)]. Other essential pathways including Amino Acid Metabolism [R2_Dry16: 624 read ($\:{log}_{10}$: 2.80)], Nucleotide Metabolism [R2_Dry16: 531 read ($\:{log}_{10}$: 2.73)], and Metabolism of Cofactors and Vitamins [R2_Dry16: 512 read ($\:{log}_{10}$: 2.71)] maintained moderate-to-high abundance levels throughout drying. The functional profiles remained remarkably consistent between processing routes R1 and R2, with R2 samples generally showing slightly higher read counts across most categories. When compared to fermentation stages, the drying phase showed approximately 4–5 fold reduction in absolute read counts across major metabolic categories, though the relative distribution of functional pathways remained stable, indicating a maintenance of core metabolic functions at a lower overall level of microbial activity during this crucial phase of cocoa processing.

Functional categories were reconstructed using the KEGG database (GhostKOALA) [40] to identify potential metabolic pathways and biological processes represented within metagenomes, these functional profiles reflect the expected metabolic and cellular potential inferred from annotation at the DNA level, rather than direct measurements of gene expression or activity. These predictions require transcriptomic or metabolomic validation. Additionally, the heatmaps were designed to illustrate relative shifts in functional potential across processing stages, rather than to imply active regulation or functional dominance of specific pathways.

In summary, a clear ecological succession was observed across the cocoa post-harvest processing, characterized by a transition from yeast-dominated communities during fermentation (*Saccharomyces*, *Pichia*) to a fungal community with a notable presence of *Aspergillus* during drying, while the bacterial population shifted from a diverse *Enterobacteriaceae* assemblage to a near-absolute dominance of *Acetobacter*. Concomitantly, the viral community exhibited a marked successional dynamic, with early-stage genera being replaced by a distinct consortium in later stages. This taxonomic restructuring was directly reflected in the functional metabolic profile, which demonstrated high activity in carbohydrate, genetic information, energy, environmental process and amino acid metabolism during early fermentation, followed by a systemic metabolic downshift in later fermentation and drying. The persistence of *Acetobacter* and the core metabolic functions throughout drying underscores their crucial role in this phase. Together, these results illustrate a tightly coupled relationship between the structure and composition of the microbial community and its metabolic potential, shaping the biochemical environment of cocoa bean processing.

## Discussion

Recent advances in high-throughput sequencing have substantially expanded the capacity to investigate food microbial ecosystems, particularly complex fermentations such as cocoa [41–44]. In this study, shotgun metagenomics was used to characterize fungi, bacterial, viral, and functional potential dynamics across fermentation and drying in two post-harvest processing routes. Importantly, the interpretations presented here are grounded in the microbial succession patterns and in KEGG-based functional potential, rather than assuming direct metabolic activity, which would require transcriptomic or metabolomic validation.

Across both routes, fermentation was consistently influenced by yeast-dominated communities, confirming the foundational role of yeasts in cocoa fermentation ecology [14, 45, 46]. However, marked route-dependent differences were observed. In Route 1, *Saccharomyces* and *Pichia* co-dominated early and mid-fermentation, whereas Route 2 showed a pronounced dominance of *Saccharomyces* (> 95%) at peak fermentation (Fig. 2). Because physicochemical variables such as temperature and pH were not measured, these differences cannot be attributed to specific abiotic drivers; instead, they reflect route-associated microbial outcomes, consistent with previous reports that spontaneous fermentations may diverge substantially depending on local processing practices [47–49].

During drying, yeast abundance declined sharply in both routes, and filamentous fungi became detectable. Notably, *Aspergillus* appeared almost exclusively during the drying phase and reached its highest abundance in Route 2 (up to 31%). Given that fermentation completion was defined by a cut test and drying by a moisture threshold (< 7.5%), these patterns suggest route-specific drying dynamics rather than uncontrolled over-fermentation. While *Aspergillus* can be part of late-stage cocoa microbiota, its increased abundance during drying underscores the importance of this phase as a critical control point for food safety [50–53]. Although mycotoxins were not measured, the presence of *Aspergillus* warrants attention, as its proliferation under suboptimal drying conditions has been linked to contamination risks in cocoa systems [50–54].

Bacterial communities exhibited a clear and reproducible successional trajectory in both routes (Fig. 3). Early fermentation stages were characterized by high bacterial diversity, dominated by *Enterobacteriaceae* and genera such as *Enterobacter*, *Salmonella*, and *Pseudomonas*, with lactic acid bacteria (LAB) present at lower abundances. As fermentation progressed, a decisive transition occurred, leading to the near-complete dominance of *Acetobacter* (> 90%), which persisted throughout drying (> 95% in all samples). This convergence toward *Acetobacter* dominance occurred in both routes despite differences in fermentation and drying duration (14 vs. 16 days), suggesting that operational endpoints rather than absolute time may be key determinants of late-stage community structure. The ubiquity of *Acetobacter* dominance aligns with extensive literature identifying this genus as a central driver of cocoa fermentation worldwide [14, 45, 55]. Importantly, although *Acetobacter* dominated both routes, earlier bacterial assemblages differed, indicating that route-specific effects are more pronounced during early fermentation, a stage that may be particularly sensitive to local practices [45, 47, 48, 56].

Bacteriophages represent a minor but notable component of the cocoa bean fermentation microbiome. Metagenomic analyses have revealed that bacteriophages comprise approximately 1% of the total microbial community during cocoa fermentation [57]. The inclusion of viral analyses provides novel insight into cocoa fermentation ecology. Some detailed phylogenetic analysis identified bacteriophage-related sequences with restricted diversity, dominated by *Myoviridae* and *Siphoviridae* families, primarily targeting *Lactobacillus* as the dominant host [58]. In this research, viral communities exhibited clear successional dynamics, transitioning from diverse early-stage assemblages dominated by *Lambdavirus* and *Punavirus* to more specialized consortia dominated by *Spbetavirus*, *Lafunavirus*, and *Pemunavirus* during late fermentation and drying (Fig. 4). Route-specific viral signatures, such as the exclusive detection of *Derbicusvirus* in Route 2 at the end of drying, further indicate that viral communities respond sensitively to processing conditions.

Although causal relationships cannot be established, the co-occurrence of dominant bacteriophages with *Enterobacter*, *Salmonella*, *Pseudomonas*, *and Acetobacter*-dominated bacterial communities is consistent with virus–host coupling observed in other fermented food systems [57, 58]. These dynamics suggest that bacteriophages may contribute to regulating bacterial populations and maintaining ecosystem stability, rather than acting as passive components of the microbiome [59]. Recent studies suggest that specific bacteriophages may contribute to improved fermentation management and food safety by influencing microbial community dynamics, while also raising the possibility of probiotic-like effects in contemporary fermented food systems, although these functions remain to be fully elucidated [60]. In addition, available evidence indicates that the diversity of bacteriophages in fermented foods may vary in relation to geographic, climatic, and environmental factors, as well as differences in raw materials, preparation methods, and underlying microbial community composition [60, 61]. However, given the limited number of cocoa fermentation studies that include viral components, these findings highlight both the novelty of this work and the need for further ecological investigation of virus–microbe interactions in cocoa systems.

KEGG-based functional profiling revealed pronounced temporal shifts in predicted metabolic potential across fermentation and drying (Figs. 5 and 6). Early fermentation stages showed high relative abundance of pathways associated with carbohydrate metabolism, amino acid metabolism, energy production, genetic information processing, and environmental information processing. These profiles reflect a microbial community with high genetic capacity for growth and substrate transformation during early fermentation, consistent with previous studies of cocoa fermentations [14, 62, 63]. As fermentation progressed, a systemic decline in the abundance of these functional categories was observed, followed by a further reduction during drying. Importantly, during drying, functional profiles converged between routes despite taxonomic differences, suggesting that environmental constraints impose similar selective pressures on metabolic potential. These patterns are consistent with reports that fermentation and drying time, aeration, and substrate availability influence microbial functional trajectories [62–66].

Crucially, these functional annotations represent potential metabolic capacity inferred from DNA-level data, not active metabolism. As such, interpretations of carbohydrate, amino acid, or energy metabolism must be regarded as hypotheses requiring transcriptomic or metabolomic validation, as previously demonstrated in integrative studies combining metagenomics, metabolomics and metatranscriptomics [14, 19, 21].

While the observed dominance of *Acetobacter* reinforces its well-established importance in cocoa fermentation [67–69], the present dataset does not support definitive identification of starter culture candidates. Taxonomic resolution did not consistently reach the species or strain level, and functional predictions alone cannot establish technological suitability. Although *Acetobacter pasteurianus* has been widely proposed as a starter candidate based on genomic and metabolic modeling studies [62, 68–70], translating metagenomic observations into starter design requires culture-dependent validation and multi-omics integration [68–70].

Similarly, while yeasts such as *Saccharomyces*, *Pichia*, and *Hanseniaspora* are known contributors to flavor development [71, 72], the present study does not resolve strain-level differences that would justify starter selection. These limitations are intrinsic to genus-level metagenomic inference and are explicitly acknowledged here.

Fermentation and drying represent critical stages for generating flavor precursors, including free amino acids and reducing sugars [19, 20, 22]. However, these processes also involve trade-offs, such as polyphenol loss and reduced antioxidant capacity [73]. While this study focuses on post-harvest microbial ecology, it is essential to recognize that roasting and Maillard reactions ultimately transform these precursors into characteristic cocoa aromas [74–77]. Thus, microbial succession during fermentation and drying sets the biochemical stage, but does not alone determine final cocoa quality.

One of the most important limitations of the study is the absence of measurement and correlation of physicochemical variables with metagenomic data. Future work integrating high-resolution physicochemical monitoring with metagenomic and metabolomic analyses would be highly valuable to further elucidate microbial dynamics during cocoa fermentation and drying.

The functional predictions based on DNA-level data should be interpreted as testable hypotheses, and the future studies integrating metatranscriptomics and metabolomics will be necessary to validate the proposed metabolic roles and bioactivities suggested by the metagenomic profiles.

Ultimately, future studies should extend beyond metagenomic potential to explore metatranscriptomics and metabolomic integration, unraveling not just which organisms and genes are present, but how and when they interact to generate flavor precursors. Moreover, the viral component of the cocoa microbiome represents an untapped area of functional relevance: understanding phage–bacteria interactions may uncover regulatory mechanisms that influence fermentation and drying stability and quality.

## Conclusions

Taken together, this study demonstrates a coordinated ecological succession involving fungi, bacteria, and viruses, accompanied by a marked shift in predicted functional potential during cocoa fermentation and drying. Despite route-specific differences in early microbial and viral communities, late-stage convergence toward *Acetobacter* dominance and reduced metabolic potential was observed. By integrating taxonomic, viral, and functional analyses, this work contributes to a more comprehensive ecological understanding of cocoa post-harvest processing, while explicitly acknowledging the methodological constraints of metagenomic inference. Future studies combining physicochemical measurements, culture-dependent approaches, and multi-omics validation will be essential to fully resolve the drivers and consequences of microbial succession in cocoa fermentation systems.

This study presents a multi-kingdom characterization of microbial community dynamics and predicted functional potential during cocoa post-harvest processing, encompassing fermentation and drying across two processing routes. Shotgun metagenomic analyses revealed a consistent successional pattern in which yeast-dominated communities (primarily *Saccharomyces* and *Pichia*) prevailed during early fermentation, followed by a transition toward acetic acid bacteria–dominated assemblages (*Acetobacter*) that persisted into the drying stage. Filamentous fungi, particularly *Aspergillus*, were detected mainly during drying, with route-dependent differences in relative abundance. In parallel, viral communities displayed structured temporal changes that broadly mirrored shifts in their potential microbial hosts.

Functional annotations based on KEGG pathways indicated marked temporal variation in metabolic potential, with early fermentation stages enriched in genes associated with carbohydrate metabolism, amino acid metabolism, energy production, and genetic information processing. As fermentation progressed and drying commenced, the relative abundance of these pathways declined, resulting in more conserved functional profiles during drying. These patterns reflect changes in the genetic capacity of the microbial communities rather than demonstrated metabolic activity, which would require transcriptomic or metabolomic validation.

From an applied perspective, the consistent dominance of *Acetobacter* during late fermentation and drying supports its well-established ecological relevance in cocoa processing. However, the increased detection of *Aspergillus* during drying emphasizes the importance of this phase as a potential control point for product safety. Although mycotoxins were not measured in this study, the observed fungal patterns underscore the need for careful management of drying conditions.

Importantly, the conclusions drawn here are constrained by several limitations. Physicochemical parameters such as temperature and pH were not measured, taxonomic resolution was generally limited to the genus level, and functional inferences were derived exclusively from DNA-level data. Consequently, implications regarding microbial activity, starter culture development, or technological optimization should be considered hypothesis-generating rather than prescriptive.

Overall, this work contributes to a more detailed ecological description of cocoa fermentation and drying by integrating bacterial, fungal, viral, and functional perspectives. Future studies combining high-resolution physicochemical monitoring with metatranscriptomics, metabolomics, and culture-dependent approaches will be essential to validate the functional roles proposed here and to establish direct links between microbial dynamics, biochemical transformations, and cocoa quality attributes. Finally, the mixed clonal composition of cocoa pods may contribute to increased microbial variability; however, this heterogeneity also enhances the ecological relevance of the study by more accurately reflecting the conditions under which cocoa fermentation occurs in real-world production systems.

## Data Availability

Accesion: BioProject PRJNA1264670 (https://www.ncbi.nlm.nih.gov/bioproject/1264670).
